# Quercetin Alleviates the Accumulation of Superoxide in Sodium Iodate-Induced Retinal Autophagy by Regulating Mitochondrial Reactive Oxygen Species Homeostasis through Enhanced Deacetyl-SOD2 via the Nrf2-PGC-1α-Sirt1 Pathway

**DOI:** 10.3390/antiox10071125

**Published:** 2021-07-14

**Authors:** Min-Yen Hsu, Yai-Ping Hsiao, Yu-Ta Lin, Connie Chen, Chee-Ming Lee, Wen-Chieh Liao, Shang-Chun Tsou, Hui-Wen Lin, Yuan-Yen Chang

**Affiliations:** 1 School of Medicine, Chung Shan Medical University, Taichung 40201, Taiwan; my.scott.hsu@gmail.com (M.-Y.H.); amy1234575@gmail.com (Y.-P.H.); yuta0301156@gmail.com (Y.-T.L.); jimboy85@gmail.com (C.-M.L.); 2 Department of Ophthalmology, Chung Shan Medical University Hospital, Taichung 40201, Taiwan; 3 Biotechnology Center, National Chung Hsing University, Taichung 40227, Taiwan; 4 Department of Optometry, Chung Shan Medical University, Taichung 40201, Taiwan; cconnie7@gmail.com (C.C.); khrnange@csmu.edu.tw (W.-C.L.); 5 Institute of Optometry, Chung Shan Medical University, Taichung 40201, Taiwan; 6 Department of Anatomy, Faculty of Medicine, Chung Shan Medical University, Taichung 40201, Taiwan; 7 Department of Nutrition, Chung Shan Medical University, Taichung 40201, Taiwan; eq7bie5d@gmail.com; 8 Department of Optometry, Asia University, Taichung 41354, Taiwan; 9 Department of Medical Research, China Medical University Hospital, China Medical University, Taichung 40447, Taiwan; 10 Department of Medical Education, Chung Shan Medical University Hospital, Taichung 40201, Taiwan

**Keywords:** age-related macular degeneration, sodium iodate, retinal pigment epithelium, quercetin, oxidative stress, autophagy, mitochondrial biogenesis

## Abstract

Oxidative damage of retinal pigment epithelium (RPE) cells plays an important role in the pathogenesis of blindness-related diseases, such as age-related macular degeneration (AMD). Quercetin, a bioactive flavonoid compound, has been shown to have a protective effect against oxidative stress-induced cell apoptosis and inflammation in RPE cells; however, the detailed mechanism underlying this protective effect is unclear. Therefore, the aim of this study was to investigate the regulatory mechanism of quercetin in a sodium iodate (NaIO_3_)-induced retinal damage. The clinical features of the mice, the production of oxidative stress, and the activity of autophagy and mitochondrial biogenesis were examined. In the mouse model, NaIO_3_ treatment caused changes in the retinal structure and reduced pupil constriction, and quercetin treatment reversed the oxidative stress-related pathology by decreasing the level of superoxide dismutase 2 (SOD2) while enhancing the serum levels of catalase and glutathione. The increased level of reactive oxygen species in the NaIO_3_-treated ARPE19 cells was improved by treatment with quercetin, accompanied by a reduction in autophagy and mitochondrial biogenesis. Our findings indicated that the effects of quercetin on regulating the generation of mtROS were dependent on increased levels of deacetyl-SOD2 through the Nrf2-PGC-1α-Sirt1 signaling pathway. These results demonstrated that quercetin may have potential therapeutic efficacy for the treatment of AMD through the regulation of mtROS homeostasis.

## 1. Introduction

Age-related macular degeneration (AMD) is a leading cause of progressive central vision loss and irreversible blindness in people older than 65 years in developed countries [1]. Nonexudative (dry) AMD is the most common subtype (approximately 90%) of all diagnosed cases, and 10–20% of these cases progress to severe exudative (wet) AMD [2]. The widespread drusen formation and RPE degeneration are the clinical features of dry AMD. The progression of AMD is attributed to a variety of risk factors, such as genetic factors, inflammatory responses, and oxidative stress [3,4]. Excessive exposure to oxidative stress caused by reactive oxygen species (ROS) of RPE cells is considered to be the leading reason in the pathophysiology of AMD [5,6,7]. Overproduction of ROS may cause a series of devastating results, including organelle damage, toxic lipoprotein debris, and extracellular drusen deposits, which eventually lead to RPE functional impairment, cell death, or dysregulated autophagy [8].

Sodium iodate (NaIO_3_), a well-known chemical oxidant, has been shown to damage retinal pigment endothelial (RPE) cells and lead to photoreceptor cell death and changes in retinal morphology [9]. The degree of RPE damage has been shown to depend on the dose of NaIO_3_; it could cause a various degree of RPE damage from the destruction of RPE function under 2–5 mM treatment, which mimics the pathogenesis of AMD [10], to the induction of RPE apoptosis and necrosis under 10 mM treatment [11,12]. Therefore, NaIO_3_-induced retinal degeneration is commonly used to study AMD in vitro and in vivo [13,14].

Autophagy is an intracellular degradation process used to remove and turn over damaged or long-lived cellular components via lysosomal degradation [15]. The impairment of autophagic processes has been demonstrated in diseases associated with oxidative stress, including malignancy, diabetes mellitus, infectious diseases, and AMD [16,17]. Moreover, the activation of autophagy often occurs during the expression of mitochondrial ROS through mTOR-dependent pathways, suggesting that autophagy can modulate the cellular antioxidative defense system [18,19]. However, autophagy plays a dual role in ophthalmic diseases. The activation of autophagy can reduce oxidative stress to preserve intracellular homeostasis, whereas the overactivation of autophagy can cause cell death [20,21].

Quercetin is a polyphenolic flavonoid compound that is found abundantly in fruits and vegetables such as onions, apples, and various berries (e.g., cranberries, chokeberries, and lingonberries) [22,23]. Previous studies have shown that quercetin is a free radical scavenger with various pharmacological applications in human health, including anti-cancer, anti-inflammatory, anti-apoptotic, antioxidant, and neuroprotective properties [24,25]. Moreover, the antioxidant effect of quercetin in RPE cells has also been demonstrated. For example, studies have found that quercetin could protect ARPE19 cells against ROS-induced cellular toxicity by increasing the total expression levels of Nrf2 (nuclear factor erythroid 2-related factor 2), thereby suppressing endoplasmic reticulum stress during superoxide-induced injury, and targeting apoptosis-associated proteins such as Bcl-2 and Bax [26,27]. In 2015, Hytti et al. suggested that quercetin avoided the oxidative stress-induced apoptosis of ARPE19 cells through the regulation of mitogen-activated protein kinase/extracellular signal-regulated kinase (MAPK/ERK) as well as cAMP-response element-binding protein (CREB) signaling pathways, accompanied by the reduction of pro-inflammatory cytokines such as interleukin-6, interleukin-8, and monocyte chemoattractant protein-1 [28]. The Nrf2/PGC-1α signaling pathway has been discussed for the regulation of mitochondrial autophagy (mitophagy) in AMD pathology [19,29]. Nrf2, as a downstream transcription factor, could substantially interact with PGC-1α to activate mitochondria-associated genes. PGC-1α was positively regulated by Sirt1, which is linked to autophagy [30]. Previous studies have also shown that Sirt1 can regulate mitochondrial ROS levels by deacetylating SOD2 [31]. Numerous studies have also clearly indicated that Sirt1 can exert antioxidative effects via the activation of Nrf2 [32]. Our previous study in 2021 also showed the protective effects of quercetin on NaIO_3_-induced oxidative stress [33]. However, the detailed mechanism about the retinal degeneration induced by NaIO_3_ and the protective effects of quercetin on NaIO_3_-induced retinal degeneration and RPE cells remains unclear. Therefore, it would be of interest to investigate whether Nrf2-PGC-1α-Sirt1 signaling plays a role in the amelioration of NaIO_3_-induced retinal damage by quercetin. Herein, the aim of the study is to investigate the protective efficacy of quercetin on the mice with AMD-like pathogenesis and to further demonstrate the molecular mechanisms of autophagy under the NaIO_3_-induced oxidative damage on ARPE19 cells.

## 2. Materials and Methods

### 2.1. Animals and Induction of Age-Related Macular Degeneration

All animal experiments were approved and conducted under the guidance of the Institutional Animal Care and Use Committee at Chung Shan Medical University (IACUC approval number: 2311). Forty-two-week-old BALB/c mice were housed in standard cages with a 12:12 h light–dark cycle. The mice were randomly divided into three groups (8 mice/group): mock, NaIO_3_-induced, and NaIO_3_ + quercetin-treated groups. The mice in the mock group were pretreated with an intraperitoneal (IP) injection of phosphate-buffered saline (PBS) followed by a single intravenous (IV) injection of PBS. The mice in the NaIO_3_ and the NaIO_3_ + quercetin-treated group received a single IV injection of 40 mg/kg NaIO_3_ [33,34]. The mice in the NaIO_3_ + quercetin-treated group were pretreated with an IP injection of 100 mg/kg quercetin before the injection of NaIO_3_. All mice were sacrificed on day 7 after performing the pupil constriction test, and samples of eyeballs were harvested for further experiments.

### 2.2. Pupil Constriction

For pupil constriction responses, dark-adapted mice were exposed to a series of illuminations (10, 50, 100, 250, 500, and 1000 l×), and images were captured under infrared illumination to measure the pupil size. An infrared light-emitting diode was used throughout the experiments for background illumination. Images were acquired using a BDPL-2 DSLR Camera (Canon, Tokyo, Japan), with a gap of at least 2 min between each measurement. The pupil area of each eye with a series of illuminations was measured using Caseviewer ver.2.3.0.99276 software (3DHISTECH, Germany). The change in pupil constriction for each mouse was calculated as the difference between the pupil area measured in the dark and in the light.

### 2.3. Histology and Immunohistochemistry

The eyeballs were enucleated and fixed in Davidson’s solution containing 10% formalin, 10% glacial acetic acid, and 4% formaldehyde for three days [33]. Paraffin-embedded sections (5 μm thick) were cut from each eye. For histological studies, the sections were stained with hematoxylin and eosin (H&E). The thicknesses of the inner nuclear layer (INL), outer nuclear layer (ONL), and the whole retina were measured along the superior and inferior hemiretina at a distance between 600 μm and 900 μm from the optic nerve. Data were obtained from six sites, and the average of each eye was used for analysis. For immunohistology, the sections were stained using a BondMax automated slide staining system (Vision BioSystems Ltd., Newcastle Upon Tyne, UK). The sections were incubated with anti-mouse LC3 antibody (G-4, Santa Cruz, CA, USA) and quantified using the Image J Immunohistochemistry Tool Box (National Institute of Health, Starkville, MS, USA). All photos were captured using an optical microscope (Olympus Optical, Tokyo, Japan).

### 2.4. Cell Culture

The human RPE cell line ARPE19 (at passage 27, product CRL-2302, American Type Culture Collection, ATCC, Manassas, VA, USA) was cultured in Dulbecco’s modified Eagle’s medium/nutrient mixture F12 Ham (HyClone, Logan, UT, USA) supplemented with 10% fetal bovine serum (Gibco) at 37 °C in a humidified atmosphere containing 5% CO_2_.

### 2.5. Cell Viability Assay

The ARPE19 cells (1.5 × 105 cells/well) were initially seeded into 24-well plates in 1 mL and cultured at 37 °C for 24 h. The culture medium was then replaced by a medium containing various doses of quercetin (0, 1.25, 2.5, 5, 10, and 20 μM) or co-treated with NaIO_3_. After 24 h of incubation, cells in each well were incubated with 0.5 mL of culture medium containing 10 μL Cell Counting Kit-8 (HY-K0301, MedChemExpress, Monmouth Junction, NJ, USA) for 1.5 h. A microplate reader (Multiskan Spectrum, Thermo Co., Vantaa, Finland) was used to measure the absorbance of each well at 450 nm.

### 2.6. Determination of Cytosolic ROS Production

Cytosolic ROS production was examined by measuring the level of fluorescent 2′,7′-dichlorofluorescein (DCF), which was oxidized from 2′,7′-dichlorofluorescein diacetate (DCFH-DA) by ROS. In brief, ARPE19 cells were cultured in 12-well plates and then pretreated with various concentrations of quercetin (1.25, 2.5, and 5 μM) for 1.5 h before incubation with 6 mM NaIO3 at 37 °C for 18 h. Culture medium was then added with 10 μM of DCFH-D at 37 °C for 30 min. After washing, the cells were collected and the mean fluorescence intensity of single cells was measured by flow cytometry (BD Biosciences, San Jose, CA, USA). Data were analyzed using CellQuest software (USA).

### 2.7. Determination of Mitochondrial ROS Production

Production of mitochondrial ROS was measured using the mitochondria-targeted red fluorogenic dye MitoSOX™ Red (Thermo Fisher Scientific, Rockford, IL, USA; cat. M36008). ARPE19 cells were cultured in 12-well plates and then pretreated with various concentrations of quercetin (0, 1.25, 2.5, and 5 μM) for 1.5 h before incubation with 6 mM of NaIO_3_ at 37 °C for 15 h. The culture medium was then aspirated, and 5 μM of MitoSOX™ Red was added for 20 min at 37 °C. After washing, the cells were stained with 2 μL of Hoechst33342 (BD Biosciences, USA), a DNA-specific fluorescent counter-staining agent, and incubated at 37 °C for 5 min in the dark. The stained cells were observed using an inverted fluorescence microscope and then collected for flow cytometry. Images of cellular fluorescence were recorded and the relative intensities of MitoSOX™ fluorescence were quantified using CellQuest Software.

### 2.8. Measurements of Antioxidative Capacities

The activities of superoxide dismutase (SOD), catalase (CAT), and reduced glutathione (GSH) were analyzed using assay kits from Cayman according to the manufacturer’s instructions (Cat. 706002, 707002, and 703002, Cayman, Ann Arbor, MI, USA). The absorbance was measured at 570 nm with an ELISA reader.

### 2.9. Western Blot Analysis

ARPE19 cells were lysed using a lysis buffer (10 mM Tris, pH 7.5 (Sigma-Aldrich Corp.), 1 mM EDTA, and 0.1% Triton X-100 (Sigma-Aldrich Corp.)). The samples were subsequently electrophoresed by 10% SDS-PAGE, followed by the transfer of separated proteins onto a PVDF membrane. The membrane was then incubated overnight with NAD-dependent deacetylase sirtuin (Sirt)-1 (sc-74504), peroxisome proliferator-activated receptor-gamma coactivator (PGC)-1α (sc-518025), heme oxygenase (HO)-1 (sc-136960), SOD2 (sc-133134), LC3B (one of the isoforms of LC3 including LC3A, LC3B, and LC3C)-II (sc-271625), p62/also called sequestosome 1 (SQSTM1) (sc-28359), and glyceraldehyde 3-phosphate dehydrogenase (GAPDH) (sc-32233) primary antibodies, which were purchased form Santa Cruz (CA, USA), or incubated with the primary antibodies from abcam (Cambridge, UK) like acetyl-SOD2 (ab214675), Bcl-2 (ab182858), and Beclin-1 (ab207612). The phopsho-Nrf2 (UH28321111) antibody was obtained from Invitrogen (Carlsbad, CA, USA). AlphaImager 2200 software (Alpha Innotech Co., San Leandro, CA, USA) was used to measure band intensities.

### 2.10. Statistical Analysis

A completely randomized design was used for the experiments. A significant difference (*p* < 0.05) among groups was tested using one-way analysis of variance (ANOVA), and differences among treatments were then tested using the least significant difference (LSD) test. All statistical analyses were performed using Statistical Analysis Software (SAS Institute Inc., Cary, NC, USA). For the Western blot statistical analysis, nonparametric tests were used. For the dependent variables, a Friedman analysis of variation (ANOVA) test was implemented with statistically significant results, re-analyzed by a Wilcoxon signed-rank test. * *p*-values ≤ 0.05 were considered statistically significant.

## 3. Results

### 3.1. Effects of Quercetin on the Retinal Histopathological Changes

**Figure 1:**
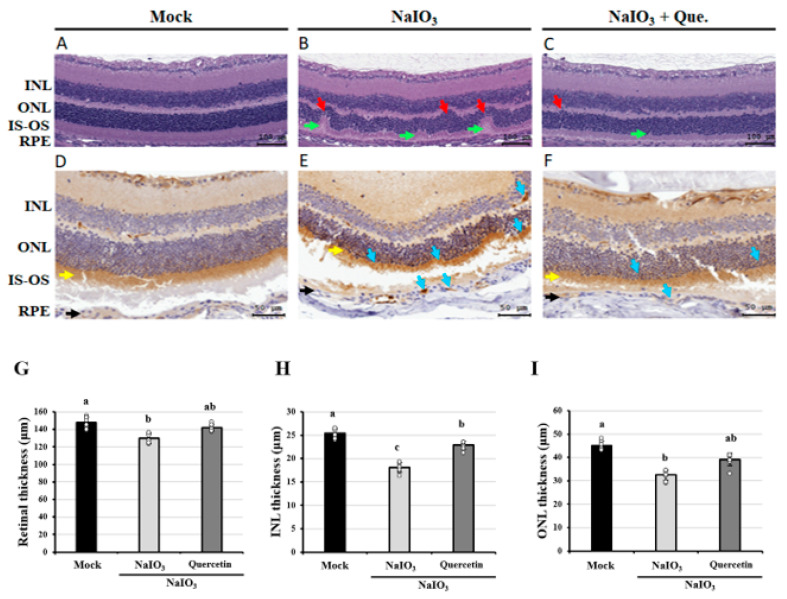
Quercetin improved the NaIO_3_-induced retinal degeneration and attenuated NaIO_3_-induced autophagy in mice retina. Eyeballs were harvested and subjected to H&E staining to assess retinal conformation and IHC staining for the expression of LC3 antibody 7 days after NaIO_3_ treatment in each group, including the (**A**,**D**) mock, (**B**,**E**) NaIO_3_, and (**C**,**F**) NaIO_3_ + quercetin-treated groups. H&E staining (**A**–**C**) showed conformational and thickness changes of the retina, inner nuclear layer (INL), outer nuclear layer (ONL) (red arrows), and inner and outer segments of the photoreceptor layer (IS-OS). Arrows in green indicate the formation of drusen-like lesions. Scale bar = 100 μm. Positive staining for LC3 (**D**–**F**) is shown in brown, and the yellow and black arrows indicate photoreceptor and RPE layers, respectively. Scale bar = 50 μm. (**G**–**I**) Retinal (**G**), INL (**H**), and ONL (**I**) thicknesses were measured at six locations and the values were then averaged. Values represent the mean ± SD (*n* = 8, 8 individual set experiments per set of groups). The letters a, b, and c indicate statistically significant difference (*p* < 0.05).

Histopathological changes in the retina, including thicknesses of the whole retina, INL, and ONL, were examined after staining with H&E. As shown in Figure 1A–C, histological evaluations of the NaIO_3_-treated mice revealed increased deformations of the whole retina, INL, and ONL compared with the mock group. Formation of drusen-like uneven lesions was found in the group treated with NaIO_3_ only. These conformational changes suggested marked disruption of the photoreceptor layer in the group treated with NaIO_3_ only. Conversely, quercetin ameliorated the deformation of the retinal layers, indicating that quercetin could effectively improve NaIO_3_-induced retinal degeneration.

Excessive activation of autophagy is associated with a wide range of diseases, including certain types of retinal cell degeneration [35]. LC3 is a major biological marker of the mammalian autophagic process, during which cytosolic LC3-I is transformed into lipidated LC3-II and incorporated into the autophagosomal membrane, leading to the fusion of lysosomes with autophagosomes to form autolysosomes and eventually degradation of the contents in autolysosomes [36]. After NaIO_3_ treatment for 7 days, tissue sections were subjected to immunohistochemical (IHC) staining for LC3. As shown in Figure 1D–F, the expression of LC3 was higher in the group treated with NaIO_3_, while quercetin showed a lower expression of LC3. Retinal, INL, and ONL thicknesses were also lower in the NaIO_3_-treated mice; however, the degeneration was almost alleviated via quercetin treatment (Figure 1G–I). Combined with increasing reports in the NaIO_3_-induced RPE cells and mice, retinal degeneration is often accompanied by the change of autophagy [37,38], thus these results indicated that quercetin may reduce the autophagy induced by NaIO_3_ treatment.

### 3.2. Quercetin Alleviated Pupillary Response Abnormalities

**Figure 2:**
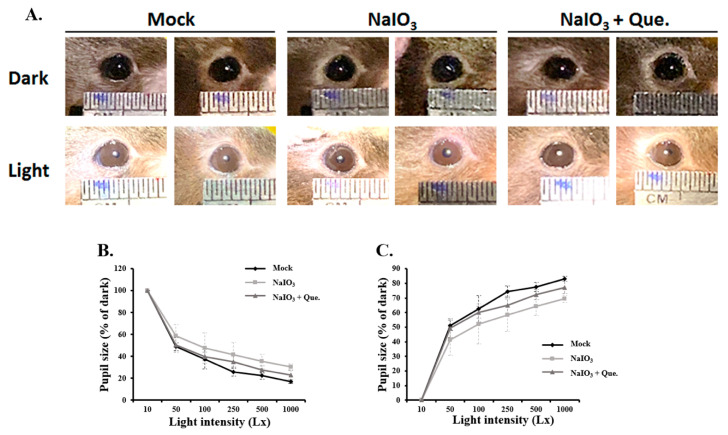
Quercetin improved the abnormal pupillary response in NaIO_3_-treated mice. Pupil sizes were measured 7 days after NaIO_3_ treatment in the mock, NaIO_3_, and NaIO_3_ + quercetin (Que.)-treated mice. (**A**) Slit-lamp pictures showed the responses of pupil size under dark/light environments. Arrows indicate the increased pupil size in the NaIO_3_-treated groups compared with the mock group (light intensity = 1000 l×). (**B**) Pupil size was measured 7 days after NaIO_3_ treatment at light intensities of 10–1000 l×, and quantified by (**C**) the percentage of pupil constriction.

Pupil constriction is a well-known measure of retinal function. In AMD patients, retina and pupil constriction are influenced by the progression of the disease [39]. To investigate the effect of quercetin on retinal degenerative changes in NaIO_3_-treated mice in vivo, a retinal degeneration mouse model was established. Pupil constriction of the mice was analyzed 7 days after NaIO_3_ treatment. As shown in Figure 2, the pupils were observed and subsequently quantified by pupil size and constriction percentage. The results showed a larger pupil size under a light background in the NaIO_3_-treated groups compared with the mock group (Figure 2A). After treatment with quercetin, the impairment in pupillary response caused by NaIO_3_ treatment improved. Pupil sizes were then measured at various background light intensities (10, 50, 100, 250, 500, and 1000 l×). In all groups, a significantly smaller pupil size was noted as the light intensity increased. At an intensity of 1000 l×, the pupil sizes in the NaIO_3_-treated groups were larger than in the mock group, and the administration of quercetin reduced the pupil size (Figure 2B). In addition, the percentage of pupil constriction significantly increased with the increase in light intensity (Figure 2C). Compared with the mock group, NaIO_3_ treatment significantly reduced pupil constriction under a light intensity of 1000 l×, and quercetin treatment ameliorated this effect. Overall, these results demonstrated that quercetin exerted a protective effect on the retina by alleviating pupillary response abnormalities in NaIO_3_-treated mice.

### 3.3. Effects of Quercetin Modulated the Activity of Anti-Oxidants on NaIO_3_-Treated Mice

**Figure 3:**
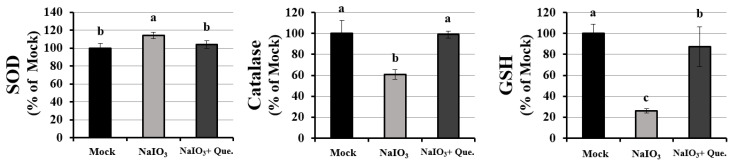
Activities of oxidative metabolic enzymes. All enzymes were measured 7 days after NaIO_3_ treatment in the mock, NaIO_3_, and NaIO_3_ + quercetin groups of mice. SOD, catalase, and glutathione were measured using commercial assay kits. Data are demonstrated as mean ± SD (*n* = 8, 8 individual set experiments per set of groups). The letters a, b, and c indicate statistically significant difference (*p* < 0.05).

In our previous studies, we showed that quercetin could attenuate the NaIO_3_-induced intracellular cell death caused by ROS by increasing catalase and GSH activity and reducing SOD activity in ARPE19 cells [33]. As cellular damage caused by ROS not only depends on the intracellular levels of ROS, but also on the balance between ROS and endogenous antioxidants, we measured the activities of catalase (CAT), glutathione (GSH), and superoxide dismutase (SOD) in NaIO_3_-treated mice. As shown in Figure 3, the expressions of CAT and GSH, but not SOD were dramatically decreased in the serum of NaIO_3_-treated mice. Quercetin treatment significantly reversed the reduced levels of SOD and the increased levels of CAT and GSH, indicating that quercetin could modulate the activity of anti-oxidants in the NaIO_3_-treated mice.

### 3.4. Quercetin Decreased the Cytotoxicity of NaIO_3_-Treated RPE Cells

**Figure 4:**
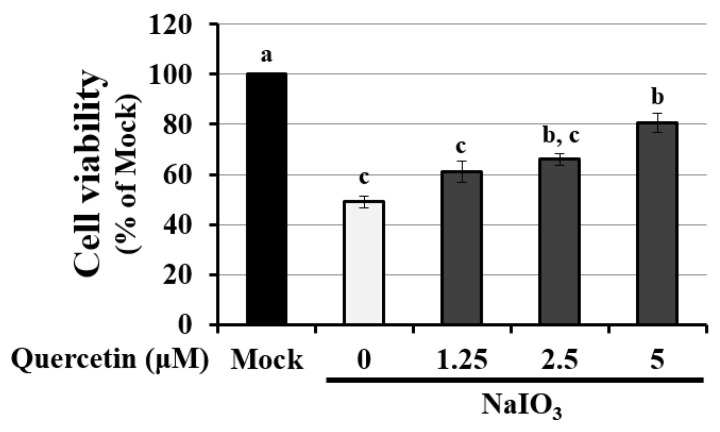
Quercetin reduced the cytotoxicity of NaIO_3_-treated ARPE19 cells. ARPE-19 cells were pretreated with 1.25–5 μM quercetin for 1.5 h, then treated with NaIO_3_ (6 mM) for 24 h. Cell viability was measured using a CCK-8 assay. Data are shown as mean ± SD (*n* = 3, 3 individual set experiments with at least two duplicates per set of groups). The letters a, b, and c indicate statistically a significant difference (*p* < 0.05).

To further investigate the detailed mechanism of quercetin on autophagy, ARPE19 cells were exposed to quercetin. The suitable concentration of quercetin was assessed according to the cell viability of ARPE19 cells pretreated with <5 μM quercetin [33]. The preventive efficacy of quercetin was assessed according to NaIO_3_-induced cell toxicity under 1.25–5 μM quercetin treatment. NaIO_3_ significantly induced cytotoxicity by almost twofold compared with the mock group, whereas quercetin treatment decreased the NaIO_3_-induced cytotoxicity in a dose-dependent manner (Figure 4). These results indicated that quercetin decreased NaIO_3_-induced cell death in ARPE19 cells.

### 3.5. Quercetin Decreased the Accumulation of ROS in NaIO_3_-Treated RPE Cells

**Figure 5:**
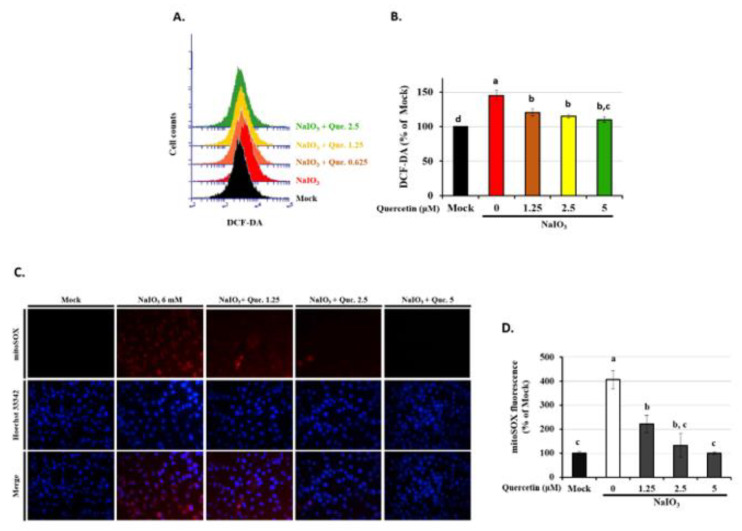
Quercetin decreased the accumulation of cytosolic and mitochondrial ROS in NaIO_3_-treated ARPE19 cells. ARPE-19 cells were either untreated (mock) or treated with 0, 1.25, 2.5, or 5 μM quercetin followed by NaIO_3_ (6 mM). (**A**) After incubation with H2DCF-DA, the results are expressed as the dot plots of DCF fluorescence per group in flow cytometry. (**C**) After incubation with MitoSOX™, the intensity of mitochondrial ROS in each group was measured using inverted fluorescence microscopic images. (**B**,**D**) The quantified analysis is shown as mean ± SD (*n* = 3, 3 individual set experiments with at least two duplicates per set of groups). The letters a, b, and c indicate statistically a significant difference (*p* < 0.05).

Quercetin, a potent antioxidant, has been reported to possess ROS scavenging capability in both in vitro and in vivo studies [33,38]. NaIO_3_ is an intracellular oxidative stress inducer, and it has been demonstrated to promote cytosolic ROS production in RPE cells. Therefore, we examined the anti-oxidative effects of quercetin induced by NaIO_3_ in ARPE19 cells. As shown in Figure 5A,B, NaIO_3_ treatment significantly induced ROS production in the cytoplasm. Pre-treatment of quercetin (1.25, 2.5, and 5 μM) significantly decreased cytosolic ROS levels induced by NaIO_3_ in a dose-dependent manner. The MitoSOX Red reagent is live-cell permeant and selectively targets mitochondria. Once in the mitochondria, MitoSOX Red is oxidized by O^2−^ and exhibits red fluorescence. The mean fluorescence intensity of mitochondrial ROS (mtROS) was significantly higher with a fourfold increase in production in the group treated with NaIO_3_ only compared with the mock group, and quercetin (1.25, 2.5, and 5 μM) pre-treatment significantly decreased the level of NaIO_3_-induced mtROS in a dose-dependent manner (Figure 5C,D). These results demonstrated that NaIO_3_ could promote not only cytosolic, but also mitochondrial ROS production, and that quercetin attenuated NaIO_3_-induced oxidative stress by suppressing both the cytosolic and mtROS levels in ARPE19 cells.

### 3.6. Quercetin Modulated Mitochondrial Biogenesis

**Figure 6:**
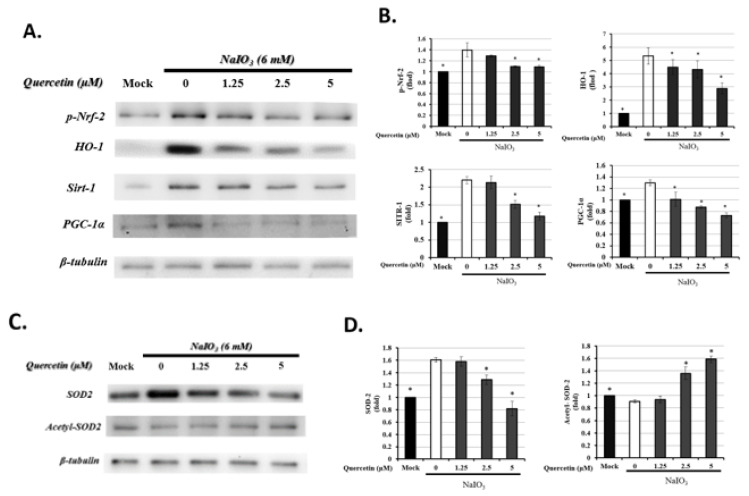
Quercetin reduced the expressions of mitochondrial proteins, including Nrf2, HO-1, Sirt1, and PGC-1α, in NaIO_3_-treated ARPE19 cells. ARPE-19 cells were either untreated (mock) or treated with 0, 1.25, 2.5, or 5 μM quercetin followed by NaIO_3_ (6 mM). Total protein from the ARPE-19 cells was extracted for the measurement of Nrf2, HO-1, SOD-2, acetyl-SOD-2, Sirt1, and PGC-1α expressions using Western blot analysis. (**A**,**C**) Representative photographs of Western blotting products are shown. The quantified expressions of (**B**) Nrf2, HO-1, SOD2, and acetyl-SOD2, as well as (**D**) Sirt1 and PGC-1α, as mean ± SD (*n* = 3, 3 individual set experiments of groups). Data were normalized to β-tubulin. * *p*-values ≤0.05 were considered statistically significant.

The Nrf2/HO-1 and Sirt1/PGC-1α signaling pathways are also responsible for the modulation of oxidative stress [40,41]. Therefore, to further determine the effect of quercetin on mitophagy, the expression of Nrf2, HO-1, Sirt1, and PGC-1α in ARPE19 cells was examined after the induction of oxidative stress. As shown in Figure 6A, the levels of p-Nrf-2 (activated state), HO-1, Sirt1, and PGC-1α were significantly higher in the NaIO_3_-treated groups than in the mock group and quercetin treatment, especially at 5 μM, and the expression levels of p-Nrf-2, HO-1, Sirt1, and PGC-1α were significantly decreased.

Li et al. reported that SOD2, an important mitochondrial oxidative scavenger, plays a key role in the regulation of mtROS, and that SOD2 activity is tightly related with acetylation at its lysine residues [32]. Therefore, we investigated the effects of quercetin-regulated NaIO_3_-induced mROS production on SOD2 expression. We used anti-acetyl SOD2 (ac-SOD2) and SOD2 antibodies to measure the level of SOD2 acetylation by Western blot, and found that, compared with the NaIO_3_-treated groups, quercetin significantly increased the acetylation level of SOD2 and inhibited the expression of SOD2 (Figure 6C).

These results indicated that quercetin could mitigate the oxidative stress induced by NaIO_3_ by influencing the acetylation of SOD2 via Nrf2/ Sirt1/PGC-1α detoxification signaling.

### 3.7. Quercetin Influenced the Expression of Autophagic Proteins

**Figure 7:**
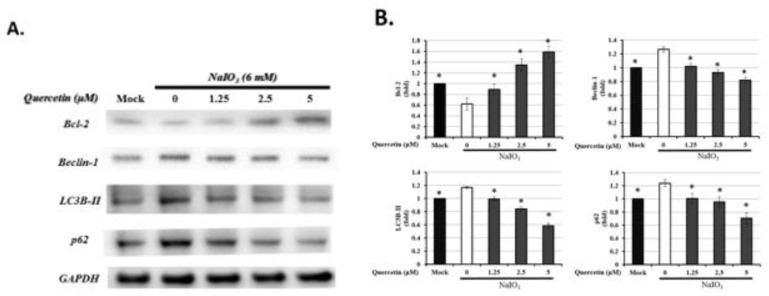
Quercetin affected the proteins related to autophagy in NaIO_3_-treated ARPE19 cells. ARPE-19 cells were either untreated (mock) or treated with 0, 1.25, 2.5, or 5 μM quercetin followed by NaIO_3_ (6 mM). (**A**) Total protein from the ARPE-19 cells was extracted for the measurement of Bcl-2, beclin-1, LC3B, and p62 expressions using Western blot analysis. The quantified expressions of (**B**) Bcl-2, beclin-1, LC3B, and p62 as mean ± SD (*n* = 3, 3 individual set experiments of groups). Data were normalized to GAPDH. * *p* values ≤0.05 were considered statistically significant.

It has been demonstrated that ROS generation can induce autophagy [18,19]. Autophagy has been reported to be involved in the oxidative responses of age-related eye diseases [21]. An increased level of oxidative stress has been shown to contribute to various autophagy-associated proteins, such as Beclin-1, Bcl-2, LC3B, and p62 [38,42]. Beclin-1 as a key autophagy inducer in mammalian cells can promote the formation of autophagosomes [43]. LC3B-II, a ubiquitin-like protein involved in autophagosome formation, is transformed from LC3B-I and localized to the membrane of autophagosomes [44]. The p62 protein can directly bind to LC3B-II during the formation of autophagosomes to eliminate the ubiquitinated misfolded proteins, and thus has also been frequently used to monitor the autophagic activities [45]. On the other hand, Bcl-2 functions as an inhibitor of Beclin-1-mediated autophagy by directly binding to Beclin-1 [46]. As shown in Figure 7, compared with the mock group, exposure to NaIO_3_ resulted in decreased Bcl-2 and increased levels of Beclin-1, LC3B-II, and p62. Treatment with 5 μM of quercetin significantly increased the level of Bcl-2 and suppressed the expression of Beclin-1, LC3B-II, and p62. These results indicated that quercetin inhibited Bcl-2-regulated cellular autophagy by downregulating the expression levels of Beclin-1, LC3B-II, and p62, and upregulating the expression level of Bcl-2 in ARPE19 cells treated with NaIO_3_.

## 4. Discussion

Autophagy is an intracellular recycling process that regulates cellular homeostasis, such as RPE cells [38,47]. However, the autophagy activity was decreased in the AMD disease, which is caused by the oxidative damage originating from the dysfunction of RPE cells [21]. Therefore, the study tried to use quercetin, a potential antioxidant substrate, to investigate the protective efficacy on the mice with retinal oxidative damage, and demonstrated the detailed mechanism in the ARPE19 cell line. In this study, quercetin improved the NaIO_3_-induced symptoms, including abnormal pupillary constriction and retinal deformation. Moreover, the conducting mechanism of quercetin was found to be accompanied by the involvement of autophagy and mitochondrial biogenesis, which showed the re-balance of mitophagy activity and the Nrf2-PCG-1α-Sirt1 signaling pathways. The present study investigated the protective efficacy of quercetin through the regulation of mitochondrial dynamics under NaIO_3_-induced oxidative damage.

NaIO_3_ has been reported to cause ROS-dependent mitochondrial dysfunction by inducing mitochondrial fission and inhibiting mitochondrial respiration [38]. Previous studies have reported that the oxidative stress caused by NaIO_3_ would selectively act on RPE cells and photoreceptors, and the clinical features on the animal models mimic those on AMD, including primary patchy loss of RPE cells followed by secondary death of not only overlying photoreceptors, but also underlying choriocapillaris [9,12], and large amounts of pigmented drusen-like deposits formed between the Bruch’s membrane and the RPE layer [48]. In our study, the NaIO_3_-induced murine model was performed to confirm the features caused by NaIO_3_. There were uneven lesions mimicking drusen in AMD pathology formed between the RPE cell layer and the ONL in the group treated with NaIO_3_ only [48,49], causing remarkable conformational changes and destruction of the photoreceptor layer, representative of oxidative wastes accumulated in the retina, indicating the successful establishment of NaIO3-induced retinal degeneration (Figure 1). Besides that, quercetin treatment exhibited therapeutic efficacy by attenuating the AMD-like features under the induction of NaIO_3_.

Mitochondria in the RPE is vulnerable to oxidative damage with aging, and thus acts as a critical organelle in the pathological processes of AMD [50]. NaIO_3_ has been proposed to dramatically induce cytosolic instead of mitochondrial ROS production in ARPE19 cells [33,38]. The excessive production of free radical species induced by NaIO_3_ leading to increased oxidative stress, represented as levels of direct superoxide generation or levels of oxidative stress markers such as superoxide dismutase, glutathione peroxidase, caspase 3, and 8-isoprostane, has been demonstrated in several previous in vivo studies [33,38,51]. Consistently, in our study, the cytosolic ROS production in the ARPE19 cells was demonstrated under the NaIO_3_-induced retinal degeneration, which was detected as hydrogen peroxide (H₂O₂) production by DCFH-DA fluorescent probes (Figure 5A,B). Additionally, we found the induction of NaIO_3_ also promoted the mitochondrial ROS as a superoxide anion (O^2−^) via MitoSOX™ Red fluorogenic dye (Figure 5C,D).

Quercetin as an effective ROS scavenger exerts anti-oxidative, anti-inflammatory, and anti-apoptotic capabilities in aging eye diseases. For example, quercetin could decrease the risk of cataractogenesis by affecting several pathways related to ocular lens opacification, mainly inclusive of oxidative stress [52]. In our study, we found that quercetin decreased the NaIO_3_-induced oxidative stress through suppression of both the cytosolic and mitochondrial ROS in ARPE19 cells (Figure 4). The results were supported by the previous study, showing that quercetin possessed the potential efficacy to prevent and delay AMD by inhibiting intracellular oxidative stress and to protect the RPE cells and the retina from NaIO_3_-mediated apoptosis [33]. However, the detailed mechanism of quercetin on retinal degeneration needed to be further investigated.

Oxidative stress is the common cellular damage upon nutrient deprivation and acts as an important mediator of autophagy, which is responsible for the elimination of detrimental proteins and organelle [53]. Therefore, autophagy has also been reported to be involved in the impaired retina of oxidative stress-related ocular diseases, such as diabetic retinopathy, glaucoma, and AMD [54]. For those associated with the present study, incomplete lysosomal degradation and increased activity of autophagy were demonstrated under the physiological condition of AMD [43]. Consistently, we found the increased expression of LC3, a standard marker of autophagy, in the NaIO_3_-induced mice (Figure 1D–F). The oxidative damage in ARPE19 cells exhibited the up-regulation of LC3B-II, Beclin-1, and p62 for the activation of autophagy, whereas the expression of Bcl-2 as the autophagy inhibitor was reduced (Figure 7). These results indicated the participation of autophagy under the NaIO_3_-induced oxidative damage.

In the present study, the cell toxicity under NaIO_3_-induced oxidative damage was improved by the treatment with quercetin (Figure 4). Meanwhile, quercetin decreased the expression of autophagy-related proteins, including LC3B-II, p62, and Beclin-1, with the induction of Bcl-2 production (Figure 7), suggesting that autophagy might be involved in the quercetin-regulated cell toxicity. Interestingly, there are some conflicting reports regarding the impact of quercetin on autophagy. The previous study applied H_2_O_2_ as an inducer to merely elevate the level of oxidative stress in ARPE19 cells, while the present study used NaIO3 to exhaustively alter the mitochondrial dynamic, including the elevated level of cytosolic and mitochondrial ROS and autophagic proteins [38]. In this study, we found that the response to NaIO_3_ treatment in mice was characterized by the overexpression of SOD2 and downregulation of CAT and GPX. SOD2, also known as manganese-dependent superoxide dismutase (MnSOD), is a mitochondrial protein that converts superoxide ions into oxygen and hydrogen peroxide. This, in turn, is transformed into water and oxygen by CAT and GSH. In contrast to SOD2, CAT and GPX levels were reduced in the NaIO_3_-treated mice (Figure 3), revealing an altered process of mitochondrial detoxification of the superoxide anions, resulting in a state of oxidative stress. This may have been caused by NaIO_3_ reducing the SOD2 acetylation level via the induction of Sirt1 expression. However, we confirmed that quercetin significantly increased the acetylation level of SOD2 and inhibited the expression of SOD2 (Figure 6C) compared with the NaIO_3_-treated group. In addition, Sirt1 has been implicated in metabolic and ROS control by regulating several antioxidant genes, such as SOD2, CAT, peroxiredoxins, thioredoxin 2, and uncoupling protein 2. These findings indicated the occurrence of mitochondrial biogenesis under NaIO_3_-induced oxidative damage.

Mitochondria act as a critical organelle for cell metabolism and the main location of autophagy occurrence [55]. The PGC-1α/Nrf2 signaling pathway has been reported to maintain cellular homeostasis under ROS-induced oxidative stress by regulating the mitochondrial biogenesis and autophagy (mitophagy), which is specifically responsible for the removal of damaged mitochondrial components [56]. This evidence pointed out that the promotion of the PGC1/Nrf2 signaling pathway accompanied by the occurrence of mitophagy was to eliminate the ROS-induced oxidative damage. However, the results were not consistent with the previous studies that proved the promotion of autophagy and the Nrf2-PGC1- Sirt1 signaling pathway accompanied by the attenuation of ROS in retinal degeneration [31,32,57]. Accumulating evidence has shown that Sirt1 plays a crucial role in cardiac protection in various cardiovascular diseases through a complex signaling network, including autophagy [58] and apoptosis [59]. Nevertheless, in our study, the upregulated expression of p-Nrf-2, PGC-1α, and Sirt1 was shown in the NaIO_3_-induced group, whereas quercetin downregulated the expression of p-Nrf-2, HO-1, Sirt1, and PGC-1α (Figure 6).

Therefore, the results in our study demonstrated that the increased production of the related proteins of autophagy and mitochondrial biogenesis was caused by the induction with NaIO_3_. The explanation is also supported by the previous studies showing that the upregulation of oxidative stress promotes the activation of mitophagy and mitochondrial biogenesis, and the cytotoxicity of RPE cells was regulated by the ROS-dependent mitochondrial dynamics [60]. Collectively, the present study results indicated that quercetin could mitigate the oxidative stress induced by NaIO_3_ by influencing the acetylation of SOD2 via Nrf2-PGC-1α-Sirt1 detoxification signaling. To the best of our knowledge, our study is the first attempt to investigate whether the action of quercetin may have potential therapeutic efficacy for the treatment of AMD through the regulation of mtROS homeostasis by deacetyl-SOD2 through the Nrf2-PGC-1α-Sirt1 signaling pathway.

**Figure 8:**
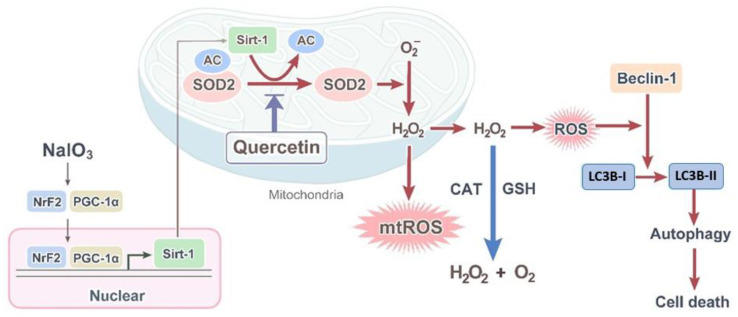
Proposed mechanism for the regulation of mtROS homeostasis by quercetin through the Sirt1 signaling pathway in NaIO_3_-induced retinal damage. Quercetin triggers the Nrf2-PGC-1α-Sirt-1 signaling pathway, which is required for Nrf2-PGC-1α-dependent Sirt1 transcription and subsequent enrichment within the mitochondria, thereby leading to deacetylation and activation of mitochondrial enzymes (SOD2) involved in mtROS regulation as well as stimulating mitochondrial H_2_O_2_ synthesis, finally contributing to mtROS homeostasis in the retina, and then alleviating NaIO_3_-induced autophagic cell death, including the upregulation of LC3B, Beclin-1, and p62, as well as downregulation of Bcl-2. Quercetin treatment decreased the levels of related factors, which controlled the quality of mitochondria. Ac: acetyl.

The present study suggested that NaIO_3_ promoted intracellular oxidative stress, including the production of cytosolic H_2_O_2_ and mitochondrial O^2−^, and was accompanied by the activation of mitochondrial biogenesis and autophagy. Moreover, to the best of our knowledge, this is the first study to investigate whether quercetin attenuated the NaIO_3_-induced retinal damage in RPE cells through mitochondrial quality control, including the reduction of mitochondrial biogenesis and autophagy (Figure 8). This evidence pointed out the potential therapeutic efficacy of quercetin on oxidative stress-induced retinal degeneration, indicating a promising strategy for the treatment of AMD through the regulation of mitochondrial activity.

## Data Availability

Data is contained within the article.
